# Simulating interaction: Using gaze-contingent eye-tracking to measure the reward value of social signals in toddlers with and without autism

**DOI:** 10.1016/j.dcn.2017.08.004

**Published:** 2017-08-12

**Authors:** Angelina Vernetti, Atsushi Senju, Tony Charman, Mark H. Johnson, Teodora Gliga, the BASIS Team

**Affiliations:** a Centre for Brain and Cognitive Development, Department of Psychological Sciences, Birkbeck, University of London, Malet Street, London, WC1E 7HX, UK; b Institute of Psychiatry, Psychology & Neuroscience, King’s College London, London, UK

**Keywords:** Social orienting, Social motivation, Unpredictability, Autism spectrum disorder, High-risk siblings, Gaze-contingency

## Abstract

Several accounts have been proposed to explain difficulties with social interaction in autism spectrum disorder (ASD), amongst which atypical social orienting, decreased social motivation or difficulties with understanding the regularities driving social interaction. This study uses gaze-contingent eye-tracking to tease apart these accounts by measuring reward related behaviours in response to different social videos. Toddlers at high or low familial risk for ASD took part in this study at age 2 and were categorised at age 3 as low risk controls (LR), high-risk with no ASD diagnosis (HR-no ASD), or with a diagnosis of ASD (HR-ASD). When the on-demand social interaction was predictable, all groups, including the HR-ASD group, looked longer and smiled more towards a person greeting them compared to a mechanical *Toy* (Condition 1) and also smiled more towards a communicative over a non-communicative person (Condition 2). However, all groups, except the HR-ASD group, selectively oriented towards a person addressing the child in different ways over an invariant social interaction (Condition 3). These findings suggest that social interaction is intrinsically rewarding for individuals with ASD, but the extent to which it is sought may be modulated by the specific variability of naturalistic social interaction.

## Highlights

• Toddlers with ASD show typical social orienting and positive facial expressions in response to predictable social stimuli.
• Variable social stimuli provoke an atypical pattern of reward-seeking behaviour in toddlers with ASD.
• Simulated social interaction, made possible by gaze-contingent eye-tracking, shows great potential to probe the conditions under which impaired social interaction emerges in ASD.

## 1 Introduction

Understanding the origin of the social interaction difficulties encountered by people with an autism spectrum disorder (ASD), whether it results from atypical *orienting* towards social stimuli, from a decreased *motivation* to engage with them, or alternatively from difficulties *understanding* and interpreting social exchanges, possibly because of their variable and complex structure, has been a key question and a challenge in ASD research (Elsabbagh and Johnson, 2016). Social orienting accounts were inspired by developmental work on neonatal face orienting abilities (Goren et al., 1975, Johnson et al., 1991 Johnson et al., 1991) and proposed that impairments in underlying cortical or sub-cortical mechanisms in ASD, would lead to decreased exposure to faces and, eventually, to cascading effects on social learning and social interaction (Elsabbagh and Johnson, 2010). Social motivation accounts expanded this view by involving reward networks and their impairment in the aetiology of ASD (Chevallier et al., 2012b). According to some authors, stimuli rich in social interactive content are best at revealing the weaker social drive in ASD. Indeed, a decreased preference for social stimuli is observed when using stimuli which depict people interacting with each other (Chevallier et al., 2015, Pierce et al., 2016, Shi et al., 2015), approaching (Crawford et al., 2016) or talking to the viewer (Dubey et al., 2015, Chawarska et al., 2013). More recently, an alternative but not exclusive theory of ASD was proposed, suggesting that social interaction difficulties may occur because of the statistical structure of such interactions. According to this account, when representing the world, individuals with ASD give too much weight to bottom-up inputs or to more recent events, to the detriment of priors computed on past events (i.e. hypo-priors, (Pellicano and Burr, 2012); low precision of prior information, (Friston et al., 2013)). One strategy for decreasing prediction error resulting from the inability to compute or give more weight to prior experience, is to preferentially engage with events that are less variable, therefore more predictable. As compared to objects driven by physical forces, interacting with human beings has a high degree of variability, both in terms of the timing and the content of responses (e.g. there are many different ways of greeting someone). Few studies have directly tested the impact that variability or predictability of an interaction has on social choices in ASD (Dawson et al., 1998). However, children with ASD exhibit more frequent social behaviours and social drive when interacting with familiar, therefore more predictable, social partners (e.g. caregivers) (Goldberg et al., 2016, Sigman et al., 1986). The decreased motivation towards social stimuli with rich interactive content documented above could also be reframed in terms of an aversion for more unpredictable stimulation.

To date, there is still little convergence within the findings to confidently support one hypothesis over another. This is partly due to the fact that many investigations into the origin of social interaction atypicalities were carried out in older children or adults. The profile of impairment in adulthood is likely to reflect idiosyncratic compensatory strategies or compounding effects resulting from a lifelong experiencing challenging social exchanges (Johnson et al., 2015). Difficulties with understanding social interactions, later in life, could be a consequence of reduced motivation to engage with others. The opposite scenario may be equally possible, difficulties with processing social cues, earlier in life, leading to decreased motivation to engage with social partners. Even when developmental populations have been considered, the paradigms employed did not always lead to conclusive interpretations. A large amount of research has measured the distribution of visual attention to scenes containing social agents or social interaction in early ASD (Chawarska et al., 2013, Elsabbagh et al., 2014, Jones and Klin, 2013, Pierce et al., 2011). For example, in, Pierce et al. (2011) 2-year-old children saw two movies displayed side by side, with one containing geometric shapes in movement and another video showing children playing. In this study, the ASD group looked less towards the social scenes than the control participants. Similarly, Jones and Klin (2013) reported a decrease in looking to people’s eyes and faces in infants with ASD from 6 months on to 2 years of age. These differences in looking time to faces and social scenes are consistent with several accounts. They could reflect an impairment in social orienting (Klin et al., 2002), but could equally result from reduced attributed reward value of social stimuli (Chevallier et al., 2012b) or from difficulties predicting when this information becomes relevant (Vivanti et al., 2011). Other studies carried out with older children and adults with ASD, using similar methodology, also fall short from teasing apart between different interpretations (Riby and Hancock, 2008, Fletcher-Watson et al., 2008).

Because social signals are increasingly considered to induce similar responses as other reward stimuli do, i.e. motivational approach as well as hedonic response (Schultz, 2006), new experimental paradigms have been developed to isolate the reward value of social signals in typical development and ASD. In Dubey et al. (2015), contrary to typical adults, adults with ASD carried out less effortful actions to see a video of a person smiling towards them as opposed to a video of a smiling person with averted gaze or a video of an object, demonstrating less approach behaviour towards social stimuli. Ewing et al. (2013) measured for how long children with or without ASD would press a key to maintain a social or a non-social stimulus on a screen (i.e. face or car), but found no group differences. Variations in the experimental design can possibly affect how sensitive these tasks are at measuring group differences. Giving participants a choice between the types of interaction, as in Dubey et al., might have exacerbated the processing of social value of the stimuli. Notwithstanding these differences, paradigms using on-demand social stimulation seem well suited to tease apart motivational from other aspects of social interaction.

In the current work, we build on the above studies to test different accounts of atypical social interaction in ASD. An interactive eye-tracking task was used to examine whether toddlers with and without ASD engaged with and appreciated different types of simulated social interaction. Participants in this study were toddlers at high-risk for ASD due to having an older sibling with the disorder. Low-risk participants had no first-degree relative ASD. About 20% of high-risk participants go on to develop ASD themselves (Ozonoff et al., 2015, Messinger et al., 2015). Another 20% will manifest subthreshold symptoms of the disorder (Messinger et al., 2013) and the remaining children will have typical development.

With the use of a gaze-contingent paradigm, toddlers had the possibility to animate one of two different videos through their gaze behaviour. Importantly, the current study manipulated both the social content and the predictable nature of the simulated interaction using different social stimuli in three different conditions. In a first condition (*Face* vs. *Toy)*, toddlers could choose between a social stimulus (a person greeting and smiling) and a non-social stimulus (a spinning musical *Toy*). In contrast to the paradigm used by Pierce et al. (2011), the stimuli were animated when the participant oriented towards them. According to the social orienting and social motivation theories, typically developing toddlers (low risk of autism) should preferably orient towards the social stimulus but toddlers with ASD should show no preference or prefer the spinning toy (Table 1). A second condition (*Towards* vs. *Away*) contrasted two social stimuli that, when looked at, displayed either a person turning and smiling towards the participant or a person turning away from the participant. According to the social motivation theory of autism, typically developing toddlers but not toddlers with ASD should preferably orient towards the more engaging social stimulus (Table 1). Finally, a third condition (*Invariant* vs*. Variable*) manipulated the variability of the social response received: an invariant interaction (showing the same clip in which a person addresses the child with Hello) was contrasted with a variable social stimulus (the person either saying Hello, Good job or smiling silently). According to the hypo-priors account, toddlers with ASD should show a preference for the invariant interaction (Table 1).

**Table 1:** Predictions based on three explanatory models: Diminished social orienting account (Klin et al., 2002), Diminished social motivation account (Chevallier et al., 2012b), Hypo-priors account (Pellicano and Burr, 2012). These accounts make different predictions about performance in this study. The symbol ‘x’ indicates the conditions under which the HR-ASD group performance would differ from the LR controls, according to the different explanatory models.

Explanatory models of atypical social attention in ASD	Condition 1	Condition 2	Condition 3
	Face vs. Toy	Towards vs. Away	Variable vs. Invariant
Diminished social orienting	x		
Diminished social motivation	x	x	
Hypo-priors (predictability)			x

## 2 Methods

### 2.1 Participants

Participants in this study were toddlers with or without familial risk for ASD, a proportion of whom were later diagnosed with ASD at age 3. 116 High-Risk (HR) participants (52 females) who had at least one older sibling with a community clinical diagnosis of ASD and 27 Low-risk (LR) participants (13 females) who had no first-degree relative with ASD enrolled in the study. All HR and LR children were full term infants (gestational ages of 38–42 weeks) recruited from a volunteer database at the Birkbeck Centre for Brain and Cognitive Development. Families attended four lab visits at 9, 15, 27 and 36 months. The experimental data reported here has been collected on a subset of these children during the 27-month visit and the clinical diagnosis was obtained during the 36-month visit (Table 2, see SOM for detailed clinical measures). Of the 116 HR enrolled in the study, 92 took part in the experiment and provided valid data (additional criteria of exclusion are explained later in this section) and also attended the 36-month visit. Experienced clinical researchers (TC, GP) reviewed information on ASD symptomatology (ADOS-2 (Lord et al., 2012), ADI-R (Lord et al., 1994), SCQ (Rutter et al., 2003)), adaptive functioning (Vineland Adaptive Behavior Scale II, (Sparrow et al., 2005), and development (Mullen Scales of Early Learning, (Mullen, 1995)) for each HR and LR child to ascertain ASD diagnostic outcome according to the DSM-5 (American Psychiatric Association, 2013) (see Supplemental Online Material (SOM) for a full description of the recruitment and diagnostic process). Of the 92 HR participants included in the analyses, 14 met the criteria for a diagnosis of ASD (hereafter, HR-ASD). The remaining 78 HR participants, without a diagnosis of ASD were classified in a HR-no ASD group. Of the 27 LR enrolled in the study, 26 took part in the experiment and provided valid data of which 24 also attended the 36-month visit. The two LR children absent in the 36-month visit were however included in the analysis as they showed typical development at the previous three visits. Recruitment, ethical approval (UK National Health Service National Research Ethics Service London REC 08/H0718/76 and 06/MRE02/73), and informed consent, as well as background data on participating families with high- and low-risk infants, were made available for the current study through the BASIS network (http://www.basisnetwork.org).

**Table 2:** Participant characteristics.

Measures	LR	HR-no ASD	HR ASD
Mean *(SD)*	N = 26	N = 78	N = 14
Gender	**12F: 14M**	**39F: 39M**	**1F: 13M**

	**Mean***(SD)*		
27-month visit			
Age (months)	**25.6***(1.1)*	**26.8***(1.5)*^c^	**26.3***(1.9)*
Mullen ELC score	**115***(14.9)*	**101***(19.0)*^c^	**85***(19.7)*^ab^
ADOS calibrated severity scores			
*- Social Affect*	**2.0***(.6)*	**2.4***(1.7)*	**4.5***(1.9)*^ab^
*- RRB*	**2.5***(2.2)*	**3.7***(2.6)*	**5.4***(1.6)*^a^

36-month visit			
Mullen ELC score	**119***(15.5)*	**105***(23.2)*^c^	**86***(29.9)*^ab^
ADOS calibrated severity scores			
*- Social Affect*	**2.5***(1.9)*	**2.6***(2.1)*	**4.1***(3.3)*
*- RRB*	**3.4***(2.3)*	**4.0***(2.6)*	**6.2***(1.6)*^ab^
ADI-R scores			
*- Social*	**.9***(1.5)*	**2.0***(2.6)*	**12.3***(5.1)*^ab^
*- Communication*	**.5***(1.1)*	**2.6***(3.4)*^c^	**12.1***(4.5)*^ab^
*- RRB*	**.1***(.3)*	**.8***(1.6)*	**5.9***(2.1)*^ab^

Abbreviations: ELC, Early Learning Composite; ADOS, Autism Diagnostic Observation Schedule; RRB, Repetitive and Restricted Behaviours; ADI-R, Autism Diagnostic Interview-Revised. Significance of pairwise comparisons: ^a^between the HR-ASD and LR groups, ^b^between the HR-ASD and HR-no ASD groups and ^c^between the HR-no ASD and LR groups.

### 2.2 Apparatus

The gaze-contingent tasks were created using MatLab (Mathworks, MA, US), the Psychophysics Toolbox extensions (Brainard, 1997, Pelli, 1997, Kleiner et al., 2007) and a custom-made eye-tracker-MatLab interface Talk2Tobii toolbox (Deligianni et al., 2011). The participants' gaze was recorded during the task via an eye tracker Tobii T120 (60 Hz sampling rate, 17-inch monitor, 1024 × 768 resolution).

### 2.3 Stimuli

**Fig. 1:**
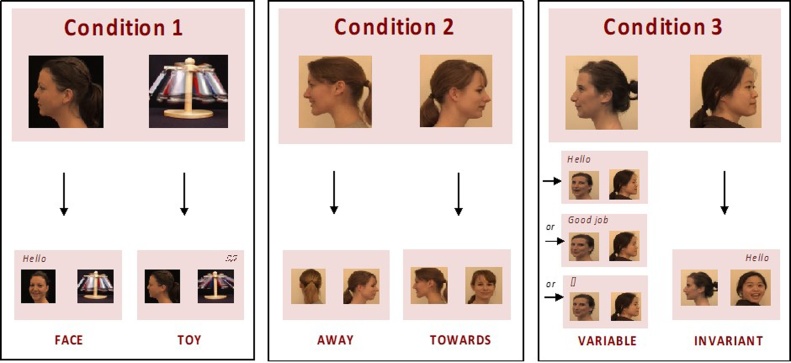
Sequence of events of a single trial for each of the three conditions. A trial started with the first frame of the two stimuli displayed on each side of a screen. Gazing at one of the two stimuli triggered the animation of the corresponding stimulus video sequence.

Three different conditions contrasting two dynamic stimuli were created (Fig. 1). In the first condition *(Face* vs. *Toy)*, one stimulus consisting of the video of a woman’s head, showing her profile and then turning towards the participant until facing the camera, at which point the addressed the participants saying ‘*hello’* with a smile (there-after stimulus *Face*). This was contrasted with a second stimulus which was a video of a metallic *To*y with brightly coloured arms rotating and accompanied by a light music of four tones (both video clips were 3.3-s-long). In the second and third conditions *(Towards* vs. *Away* and *Invariant* vs. *Variable)*, the two stimuli consisted of the videos of two different women. In the second condition, a woman turned her head towards the participant and smiled (there-after stimulus *Towards*) while the other woman turned her head away from the participant until the back of her head was visible (there-after stimulus *Away)* (both video clips were 3.3-s-long). In the third condition, a woman always turned her head towards the participants and said ‘*hello*’ (there-after stimulus *Invariant)* while another woman turned her head towards the participant and either smile silently (1.6-s-long), said ‘*hello’* (2.5-s-long) or said ‘*good job*’ (2.9-s-long) (there-after stimulus *Variable*). Only the women’s heads were visible. Different actresses were used in each condition (one identity in Condition 1, and 2 new identities in each of Condition 2 and 3, see Fig. 1), thus 5 different identities were used throughout the study.

### 2.4 Task

The task consisted of the presentation of two dynamic stimuli displayed on the left and right side of the screen (350 × 400 pixel each) in the three different conditions. In each condition, a trial started with a central fixation (a 75 × 75-pixel animation of a spinning ball), which disappeared when gaze was detected within an area covering it. The first stimulus screen depicted still images of the two stimuli. When the participants gazed at one of the two stimuli for 100 ms, this triggered the video sequence of the corresponding stimulus. For all the three conditions, only one video of the two stimuli was triggered and displayed in each trial. If the participant shifted his/her gaze towards the second stimulus, this did not trigger the corresponding video clip. Thus, the length of each trial was determined by the amount of time the participant would take to gaze to one of the two stimuli plus the length of the corresponding video. Finally, if the participant did not look at any of the two stimuli presented within 5 s from the beginning of the trial, the trial was terminated (and considered invalid), and a new fixation stimulus was presented. To familiarize the participant with the different available stimuli, the first trials were not gaze-contingent and consisted of the automatic presentation of the video of the two stimuli. In condition 1 *(Face* vs. *Toy)*, the first two trials of each block consisted in the presentation of the *Face* and *Toy* stimuli (the order of presentation was randomised in each block). In condition 2 *(Towards* vs. *Away)*, the first two trials of each block consisted of the presentation of the *Towards* and the *Away* stimuli (the order of presentation was randomised in each block). For both conditions 1 and 2, the remaining 6 trials of each block were gaze-contingent and the videos of the stimuli were displayed with a delay of 500 ms upon the participant’s gaze being detected on one of the two stimuli. In condition 3 *(Invariant* vs. *Variable),* the second and fourth trials were not gaze-contingent and consisted of the automatic presentation of the *Variable* stimuli, all other trials were gaze-contingent. Each block of the *condition 3* contained 17 trials. To further increase the variability of the *Variable* stimulus, the video clip was displayed after a random delay of 0–1000 ms while the *Invariant* stimulus was always presented after a 500 ms delay.

### 2.5 Procedure

Infants were seated in their caregiver’s lap, at approximately 60 cm from the screen. The task, was embedded in a longer eye-tracking testing session. Each condition consisted of 2 blocks to counterbalance the left or right location of the two types of stimuli on the screen (i.e. the stimulus *Face* from the condition 1 was presented on the left side in the first block; the stimulus *Towards* from the condition 2 was presented on the right side in the first block; finally, the stimulus *Invariant* from the condition 3 was presented on the left side in the first block). All the participants were given the same order of presentation: condition1-block1, condition1-block2, [other tasks], condition3-block1, condition2-block1, [other tasks], condition2-block2, condition3-block2.

### 2.6 Measures

We coded two key variables to assess the assignment of reward value to the stimuli by the participants. Firstly, we coded an eye-tracking measure, *initial looks*, which is the first look that the participants made towards one of the two stimuli at the beginning of each trial. This measurement has been used in previous gaze-contingent reinforcement paradigms in human infants and children (Wang et al., 2012, Vernetti et al., 2017), as an index of reward-seeking behaviour in the same way that persistent manipulation of touch-panel (Dubey et al., 2015) or keyboard (Ewing et al., 2013) has been used for older children. More frequent *initial looks* indicate a higher incentive value of the stimuli, or infants ‘want' it more (Chevallier et al., 2012b). Secondly, we coded the frequency of *smiles,* which is the participant's positive facial expression in response to their first choice of stimuli. This is an index of hedonic response (Berridge, 2004, Smith et al., 2011), or whether infants ‘liked' the stimuli (Chevallier et al., 2012b). Interestingly, hedonic responses to social interaction have scarcely been measured in ASD, but existing evidence suggests a developmental decrease in social smiling in high-risk siblings from 6 to 36 months of age (Ozonoff et al., 2010) as well as lower reported pleasure of social interaction in adults (Foulkes et al., 2014, Chevallier et al., 2012a). The frequency of *smiles* was coded and derived as follows. For each trial, the participants' facial expressions were coded by an experimenter, who was blind to the study hypotheses, of the following categories: *neutral*, *smile*, *face covered*, *fussing*, *looks away soon after choice*, *parental interference*, *recording non-available*. All codes apart from *neutral* (code 0) and *smile* (code 1) were re-coded as invalid/missing data. Data from 13 participants (11%) of participants was double coded by one of the authors (TG). There was good agreement between the coders (0/1/missing: Kappa = 0.707, 0/1 Kappa = 0.810).

Two additional measures (*looking time* and *second looks*) were computed. The *looking time* was the cumulative time the participants spent looking at the two different stimuli. The *second looks* corresponded to the participants' reorienting of gaze towards the opposite stimulus within two seconds after the *initial looks*. The *second looks* are a complementary index of reward-seeking behaviour, and more reorienting from a less rewarding stimulus to a more rewarding stimulus was predicted, mirroring the *initial looks*. Since the analysis of *second looks* were similar to the analyses of *initial looks*, the results of this analysis are reported in the SOM.

The gaze behaviour and hedonic responses were analysed with Generalized Estimating Equation (GEE) analyses that were built with a binomial distribution, a logit link function, an unstructured correlation matrix and a robust estimator. Bonferroni corrections were applied to the post-hoc analyses. Preliminary analysis revealed a strong leftward side bias in the initial looks measure (see SOM), therefore only the participants contributing valid trials from both blocks in each condition were included in the analyses (25 LR and 90 HR in condition 1, 21 LR and 80 HR in condition 2 and 21 LR and 81 HR in condition 3).

Additional analysis accounted for the fact that some of the participants were taking part in an intervention programme, involving video-mediated parental training (Green et al., 2015, Green et al., 2013). Although this intervention did impact on symptom severity and social attention, there was no significant difference between the treated (n = 27) and non-treated (n = 25) groups in terms of clinical outcome. Thus, the distribution of the treated children in the different outcome groups was not affected by the intervention. However, to rule out any effect of the recruitment or intervention on social orienting in our study, we also report the GEE results after including the factors Treatment and Recruitment (for intervention) in the SOM. Follow-up analyses including these factors did not change the significance level of the effects of interest reported in the results section (see SOM for further details).

The first 2 trials in Condition 1 and 2 (non-contingent) and the first 4 trials in Condition 3, were not included in analyses. There were no significant differences in the number of valid trials (i.e. trials in which a video of a stimulus was triggered by the participants’ gaze) included in the analyses of *initial looks* and *looking time* between the different Outcome groups, in either condition (*Condition 1*: F (2, 114) = 0.66, p = 0.519; *Condition 2:* F (2, 100) = 0.94, p = 0.394; *Condition 3:* F (2, 101) = 0.04, p = 0.958, Table 3). There were also no significant differences in the number of valid trials included in the analyses of *smiles* between the different Outcome groups, in the *Condition 2* (F (2, 62) = 0.01, p = 0.995) and *Condition 3* (F (2, 63) = 0.12, p = 0.888, Table 3). However, in the *Condition* 1, the LR group had a smaller number of valid trials than the HR-no ASD and HR-ASD groups (*F* (2, 66) = 8.66, p < 0.001). Nevertheless, no significant correlation between the proportion of smiles and the average number of valid trials was found in the Condition 1 (r = 0.09, p = 0.464).

**Table 3:** Number of valid trials used for the analyses of Initial first looks, Looking time and Smiles, for each condition (1: Face vs. Toy; 2: Towards vs. Away; 3: Variable vs. Invariant) and each outcome group (LR, HR-no ASD, HR-ASD). ^*^Number of valid trials out of 12 total trials for condition 1 and 2 and out of 26 total trials for condition 3. (Average number of valid trials, *SD* and Number of participants).

Valid trials	Condition	LR		HR-no ASD		HR-ASD	
		**Mean***(SD)*	N	**Mean***(SD)*	N	**Mean***(SD)*	N
Analyses of Initial first looks and Looking time	Condition 1. Face vs. Toy	**11.6***(.6)*	25	**11.6***(.8)*	76	**11.9***(.4)*	14
	Condition 2. Towards vs. Away	**11.7***(.6)*	21	**11.3***(1.2)*	66	**11.4***(1.3)*	14
	Condition 3. Variable vs. Invariant	**24.1***(3.1)*	21	**24.3***(2.2)*	67	**24.4***(2.4)*	14

Analyses of Smiles	Condition 1. Face vs. Toy	**8.4***(3.0)*	14	**10.8***(1.6)*	43	**10.9***(1.9)*	10
	Condition 2. Towards vs. Away	**8.9***(2.6)*	14	**9.0***(2.4)*	39	**9.0***(2.4)*	10
	Condition 3. Variable vs. Invariant	**21.5***(4.3)*	14	**20.8***(5.4)*	40	**21.4***(4.7)*	10

## 3 Results

### 3.1 Initial looks

#### 3.1.1 Chance level comparisons

**Fig. 2:**
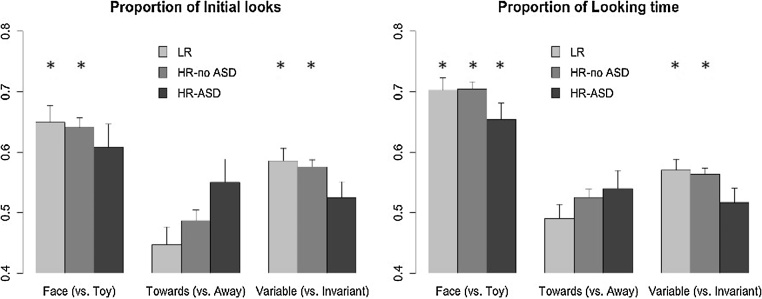
Proportion of *initial looks* and *looking time* (marginal means from the GEE analysis) towards the stimulus *Face* (Condition 1, left panel), *Towards* (Condition 2, central panel) and *Variable* (Condition 3, right panel). The proportion of *initial looks and looking time* are plotted for each group Outcome. ^*^Significance of pairwise comparisons of the proportion of *initial looks* against chance level. Error-bars: +/− 1 standard error.

To assess whether the participants preferentially oriented towards the stimuli *Face* (condition 1), *Towards* (condition 2) and *Invariant* (condition 3), the proportion of *initial looks* towards these stimuli, averaged across the two blocks were entered in one sample *t*-tests against chance level, for each condition separately (Fig. 2). In Condition 1, the analyses revealed that in contrast to the LR (t (24) = 3.59, p = 0.001, d = 0.72) and HR-no ASD (t (75) = 5.20, p < 0.001, d = 0.60) groups, the HR-ASD group did not gaze at the stimuli *Face* significantly above chance level (t (13) = 1.74, p = 0.105, d = 0.47). In Condition 2, none of the groups preferably gazed at the stimulus *Towards* (all p > 0.139). Finally, in Condition 3, in contrast to the LR (t (20) = 2.57, p = 0.018, d = 0.56) and HR-no ASD (t (66) = 4.18, p < 0.001, d = 0.51), the HR-ASD group did not preferably gaze at the *Variable* stimulus (t (13) = 0.68, p = 0.507, d = 0.18).

#### 3.1.2 GEE analysis of initial looks

To evaluate the participants’ visual orientation towards one of the two stimuli of each condition over the course of the task, the *initial looks* were entered in a GEE analysis. The stimuli *Face* (Condition 1), *Towards* (Condition 2) and *Variable* (Condition 3) were coded as 1 while the other corresponding choices were coded as 0. Trials, Block were entered in the model as within-subject factors and Outcome (LR, HR-no ASD, HR-ASD) as a between-subject factor. The analyses revealed a main effect of Outcome (Waldχ^2^(2) = 24.23, p < 001 with HR-ASD making more pro-social choices than the LR (p = 0.001) and HR-no ASD (p < 0.001). There was also a main effect of Condition (Waldχ^2^(2) = 35.63, p < 0.001, with Condition 2 receiving the least pro-social choices, significantly less than Condition 1 and 3 (both p < 0.001). There was no significant difference between these last two conditions (p = 0.285). Finally, two significant 2-way interactions between Outcome and Condition (Waldχ^2^(4) = 15.16, p = 0.004) and Block and Condition (Waldχ^2^(2) = 26.60, p < 0.001) were found as well as a marginal 3-way interaction between Outcome, Condition and Block (Waldχ^2^(4) = 9.21, p = 0.056). We followed-up on this interaction with three GEEs, one for each condition. Main effects of Outcome were reported first and follow-up analyses of main effects of Block and of 2-way interaction between Outcome and Block are presented last, as there were not the main effects of interest. *Condition 1: Face vs. Toy*. The analyses revealed no significant effect of Outcome (Waldχ^2^(2) = 0.06, p = 973). The analyses also revealed significant effects of Trials (an increase in *initial looks* towards the stimulus *Face* over trials, Waldχ^2^(1) = 10.17, p = 0.001) and Block (a decrease in *initial looks* from Block 1–2, Waldχ^2^(1) = 42.75, p < 0.001) as well as an interaction between Outcome and Block (Waldχ^2^(2) = 6.05, p = 0.048), driven by a decrease in *initial looks* from Block 1–2 for the LR (p = 0.001) and HR-no ASD (p < 0.001) groups but not for the HR-ASD group (p = 1.000). *Condition 2: Towards vs. Away*. The analyses revealed a significant effect of Outcome (Waldχ^2^(2) = 6.07, p = 0.048) which was driven by a higher proportion of *initial looks* directed to the *Towards* stimulus for the HR-ASD group than for the LR group (p=0.038). Significant effects of Trials (a significant decrease in *initial looks* towards the stimulus *Towards* (Waldχ^2^(1) = 6.82, p = 0.009) and Block (an increase in *Towards* looking from Block 1–2, Waldχ^2^(1) = 29.68, p < 0.001), were also observed, but there was no significant interaction between Outcome and Block (Waldχ^2^(2) = 1.05, p = 0.591). *Condition 3: Variable vs. Invariant*. The analyses revealed a non-significant main effect of Outcome (Waldχ^2^(2) = 0.91, p = 0.634). There was no significant main effect of Trials (Waldχ^2^(1) = 1.54, p = 0.214) but a significant effect of Block (a decrease in *initial looks* towards the stimulus *Variable* from Block 1–2, Waldχ^2^(1) = 22.32, p < 0.001) as well as a significant interaction between Outcome and Block (Waldχ^2^(2) = 8.12, p = 0.017) which was driven by a higher proportion of prosocial choices for the LR group compared to the HR-ASD group in the first block (p = 0.010) and by a decrease in *initial looks* from Block 1–2 for the HR-no ASD group (p < 0.001).

### 3.2 Looking time

#### 3.2.1 Chance level comparisons

The proportions of looking time towards the *target* stimuli *Face* (Condition 1), *Towards* (Condition 2) or *Variable* (Condition 3) compared to the *non-target* stimuli *Toy* (Condition 1), *Away* (Condition 2) or *Invariant* (Condition 3) were computed as follow: “looking time towards *target*/(looking time towards *target* + looking time towards *non*-*target*)”. The proportion of *looking time* were then entered in one sample *t*-tests against chance level of 0.5, for each condition separately (Fig. 2). *Condition 1: Face vs. Toy*. All groups, including the group HR-ASD looked longer towards the stimulus *Face* compared to the *Toy* (all t > 3.13, p < 0.008, all d > 0.84). *Condition 2: Towards vs. Away*. None of the groups spend more time looking at either of the *Towards* or *Away* stimuli (all t < 1.61, all p > 0.0112, all d < 0.20). *Condition 3: Variable vs. Invariant*. Both the LR and the HR-no ASD groups looked longer towards the stimulus *Variable* compared to the *Invariant* stimulus (both t > 2.84, both p < 0.010, both d > 0.62). This was not the case for the HR-ASD group (t (13) = 0.72, p = 0.484, d = 0.19). Overall, the measure of *initial looks* and the measure of looking time seem to reflect the participants’ first selection. The participants spent more time looking at the stimulus their first gazed at. Indeed, these two measures were found to be highly correlated in all three conditions (*Condition 1*: r (115) = 0.94, p < 0.001; *Condition 2*: r (101) = 0.78, p < 0.001; *Condition 3*: r (102) = 0.94, p < 0.001).

#### 3.2.2 Comparisons of proportions of looking time between groups

A mixed ANOVA examining the proportion of looking time with the group Outcome as between-subject factor and the factor Condition (*Face* vs. *Toy*, *Towards* vs. *Away*, *Variable* vs. *Invariant*) as within-subject factor was also conducted to examine any differences of looking time between the three conditions. No main effect of Outcome nor interaction between Outcome and Condition were found (both F < 0.67, both p > 0.513). However, a main effect of Condition was found (F (2, 220) = 19.83, p < 0.001, η_p_^2^ = 15). This effect was driven by a higher proportion of *looking time* towards the *Face* (vs. *Toy* in Condition 1), compared to the stimulus *Towards* (vs. *Away* in Condition 2, p < 0.001) and the stimulus *Variable* (vs. *Invariant* in Condition 3, p < 0.001). No differences in proportion of *looking time* towards the prosocial stimuli (*Face*, *Towards* and *Variable*) between the Conditions 2 and 3 were found (p = 0.296).

### 3.3 Smiles

**Fig. 3:**
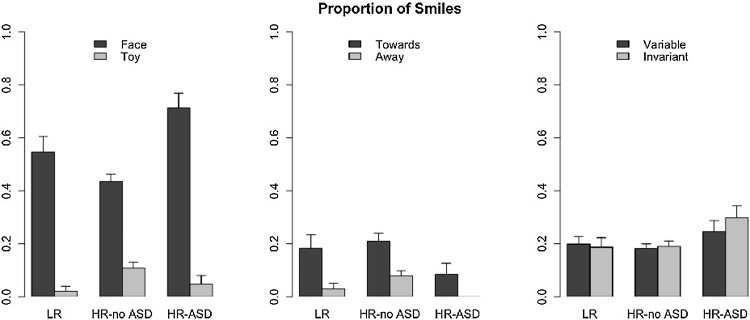
Proportion of *smiles* (marginal means from the GEE analysis) towards the two stimuli in each condition. Condition 1 (left panel), Condition 2 (central panel) Condition 3 (right panel). The proportion of *smiles* is plotted for each group Outcome. Error-bars: +/− 1 standard error.

To evaluate the participants’ hedonic response towards the stimuli of each condition, positive facial expressions categorised as *smiles* were entered in a GEE analysis (Fig. 3). A smaller number of participants were included in the analysis of *smiles* (n = 74) due to the difficulty to code facial expression of the participants in the video recordings or due to missing video recordings. A description of the characteristics of this sample is reported in the SOM to show that they were representative of the whole group. Condition, Trials and Block were entered as within-subject factors and Outcome as between subject factor in the model. The Type of stimulus that was triggered on each trial (*Face*, *Towards* and *Variable* vs. *Toy, Away* or *Invariant*) was also included as a within-subject factor in the model. This analysis yielded a significant effect of Condition (Waldχ^2^(2) = 57.11, p < 0.001), with most *smiles* in Condition 1 and least *smiles* in Condition 2 (all pairwise p < 0.016) and a marginal effect of Outcome (Waldχ^2^(2) = 5.54, p = 0.063). There was also a main effect of the Type of stimulus, with the prosocial stimuli (*Face*, *Towards* and *Variable*) eliciting more *smiles* (Waldχ^2^(1) = 39.07, p < 0.001). Finally, two significant interactions between Outcome and Condition (Waldχ^2^(4) = 33.06, p < 0.001) and between Condition and Type of stimulus (Waldχ^2^(2) = 89.17, p < 0.001) were found. We followed-up on the significant interaction between Outcome and Condition with three GEEs, one per condition. *Condition 1: Face vs. Toy*. The analyses revealed no significant effect of Outcome (Waldχ^2^(2) = 5.15, p = 0.076) but a significant effect of Type of stimulus, showing more *smiles* towards the stimulus *Face* than the stimulus *Toy* (Waldχ^2^(1) = 58.97, p < 0.001). There was also a significant 2-way interaction between Outcome and Type of stimulus (Waldχ^2^(2) = 14.05, p = 0.001). When examining the proportions of *smiles* separately for the stimuli *Face* and *Toy*, this yielded an effect of Outcome for the stimulus *Face* (Waldχ^2^(2) = 6.24, p = 0.04), driven by a higher proportion of *smiles* from the HR-ASD group than the HR-no ASD group (p = 0.015). Outcome groups did not differ in the proportion of *Toy* choice trials eliciting smiling (Waldχ^2^(2) = 4.73, p = 0.09). There were no significant effects of Block (Waldχ^2^(1) = 2.28, p = 0.131) or Trials (Waldχ^2^(1) = 0.06, p = 0.814). *Condition 2: Towards vs. Away*. The analyses revealed a marginal effect of Outcome (Waldχ^2^(2) = 5.80, p = 0.055). The HR-ASD group smiled less overall than the HR-no ASD group (p = 0.008). There was also a significant main effect of Type of stimulus which was due to the participants smiling more in response to the stimulus *Towards* than the stimulus *Away* (Waldχ^2^(1) = 23.72, p < 0.001). Two way interactions could not be computed due to quasi-complete separation, which reflects both the small sample going into each group and the very low rates of smiling in this condition, with HR-ASD all scoring zero when the *Away* stimulus was triggered. No significant effects of Trials (Waldχ^2^(1) = 1.47, p = 0.225) or Block (Waldχ^2^(1) = 1.00, p = 0.316) were found. *Condition 3: Variable vs. Invariant*. The analyses revealed a significant effect of Outcome (Waldχ^2^(2) = 7.15, p = 0.028). Post hoc analyses revealed that on the whole, the HR-ASD smiled significantly more than the HR-no ASD group (p = 0.008). There was no effect of Type of stimulus (Waldχ^2^(1) = 0.08, p = 0.784), no significant 2-way interaction between Outcome and Type of stimulus (Waldχ^2^(2) = 2.30, p = 0.316). There was no effect of Trials (Waldχ^2^(1) = 1.13, p = 0.288) but a main effect of Block (a decrease in smiles from Block 1–2, Waldχ^2^(1) = 4.76, p = 0.029).

## 4 Discussion

Young children with ASD encounter difficulties in interacting in social contexts from early on, and this aspect is a central characteristic used in the diagnosis of ASD (American Psychiatric Association, 2013, Constantino and Charman, 2016). Recent studies with young infants with ASD have shown that attention towards social stimuli like faces and eyes might be present during the first few months of life (Elsabbagh et al., 2013) but decline later on (Jones and Klin, 2013). To explain atypical engagement with social information in ASD, several non-exclusive theories have proposed a diminished motivation towards social stimuli (Chevallier et al., 2012b) and a difficulty to use prior information to guide behaviour, leading to a preference for more-predictable, non-social events (Chevallier et al., 2012a, Pellicano and Burr, 2012). This study aimed to tease apart these accounts by examining how different aspects of social stimulation can modulate reward-related behaviours such as motivational approach, persisting behaviour and hedonic response. More specifically, the three different conditions employed in this study tested whether the social, communicative and variable nature of the stimuli would elicit preferential orienting as well as hedonic responses from toddlers with or without ASD.

### 4.1 Capturing social reward in typical development

Altogether, preferential *initial looks* and *looking time* were observed for the *Face* (vs. *Toy*) and *Variable* (vs. *Invariant*) stimuli but to a lesser extent for the stimulus *Towards* (vs. *Away*). A preference for orienting and maintaining attention to the social stimulus *Face* over the *Toy* was observed in typically developing toddlers (LR-group). Attentional biases to orient towards face-like stimuli could explain such preference since the two stimuli were visually dissimilar from the onset of the task (Johnson et al., 1991). Additional measures from this task suggest that social orienting is not the sole driver of these preferences. Firstly, orienting towards the social stimulus *Face* (vs. *Toy*) increased over the course of the task. This preferential orienting was supported with the analyses of *second looks* (see SOM) showing that typical toddlers also shifted more their attention towards the *Face* after an initial gaze at a very salient spinning *Toy* than the reverse. Secondly, toddlers also smiled more towards this stimulus compared to the *Toy,* suggesting that the *Face* is assigned positive hedonic value (Berridge et al., 2009).

No systematic preferences were recorded in the second condition contrasting persons looking toward or away from the typically developing toddlers (LR) participants. A decrease in gazing at the stimulus *Towards* was observed throughout the task. In this condition, toddlers gradually oriented their attention to preferentially activate the simulated interaction looking *Away*. This behaviour was not predicted but could result from the lesser engaging content of the stimulus *Towards* which did not contain any auditory components (i.e. silent smile), unlike the social stimuli in the other two conditions to which the toddlers were exposed. Indeed, in another study, infants look longer to faces with infant directed speech than silent faces (Kim and Johnson, 2014). The *Away* stimulus was also more novel, amongst the higher frequency of pro-social interaction, in all conditions. However, toddlers directed more *smiles* towards the stimulus *Towards*, suggesting that, when experienced, this particular communicative interaction was assigned higher hedonic values, or they ‘liked' it more.

In the third condition, the *Variable* interaction was preferably looked at over the *Invariant* stimulus and preferences for the *Variable* stimulus remained stable throughout the task suggesting that the four initial trials were sufficient to induce preferential orienting in typically developing toddlers (LR group). In the absence of a control condition contrasting *Invariant* and *Variable* non-social stimuli, these biases towards the *Variable* stimulation cannot be assumed to be specific to social stimuli. However, the social *Invariant* stimulus was very similar to the social stimulus (*Face*) used in the first condition, which implies a hierarchy of preferences in which a *Variable* social stimulus elicits preferential orienting over an *Invariant* social stimulus, in turn preferred to a non-social stimulus. At the same time, the *Variable* stimulus did not elicit more frequent *smiles*; thus, the preferential orienting to the *Variable* stimulus might not be driven by stronger hedonic value per se.

Across all conditions, this task appears to successfully capture social reward in populations that cannot express preferences verbally or using manual choices. Moreover, we succeed at characterising both incentive and hedonic aspects of reward, thus providing a fuller picture of toddler’s engagement with social stimulation.

### 4.2 Outcome group differences

This study aims to tease apart three accounts of atypical social interaction in ASD. The current findings argue against atypical social orienting or social motivation, in toddlers at high-risk for ASD who receive an ASD diagnosis. Although the HR-ASD group, unlike the other three outcome groups, did not show significantly more frequent *initial looks* to the *Face* than the *Toy* stimuli, the GEE analysis comparing the different Outcome groups failed to show significant differences in the *initial looks*. Moreover, just like the other groups, HR-ASD showed longer *looking time* and more frequent *second looks* towards the *Face* stimulus (see SOM). HR-ASD also smiled more towards the *Face* stimulus than the *Toy* and smiled more towards the *Face* than the other at-risk group did. Thus, the current study contrasts with previous findings, in which toddlers with ASD showed more preferential looking towards geometric patterns as opposed to dynamic social scenes (Pierce et al., 2011, Pierce et al., 2016). In Pierce et al., toddlers’ preference for geometric stimuli was associated with lower developmental functioning (lower Mullen scores) and higher autistic traits (higher ADOS scores), while no such associations were found in this current study (additional analyses reported in SOM). However, we have to note that our HR-ASD group scored in average 85 on the Mullen Early Learning Composite, compared to only 77, in Pierce et al. It remains therefore possible that decreased social orienting and enjoyment is present in populations with lower IQ. The differences observed between the two studies may also come from the nature of the experimental design. In this current study, toddlers had control over the visual stimulation received, the timing and the content of the stimulation was predictable, in contrast to the social content displayed in Pierce et al. (2011). Only a few studies have given participants with ASD control over visual stimulation. In one particular study, detecting mutual gaze improved if the participants with ASD could adjust the gaze direction themselves via the use of a joystick (Dratsch et al., 2013). Some have suggested that it is the multi-modal content of social interaction that HR-ASD infants may dislike. Shic et al. (2014) showed decreased looking towards an actress face when she started addressing the infant. Yet, in our study, all groups, including HR-ASD directed more first looks, looking time and smiling towards the person addressing them with a smile and “*hello*”, versus a toy, in Condition 1, but also more than towards a person addressing them with a silent smile, in Condition 2. Again, the difference may lie in the fact that the interaction, including the onset of speech, was predictable in conditions 1 of our study.

In Condition 2, as mentioned above, despite not triggering preferential orienting, the stimulus *Towards* elicited more *smiles* than the stimulus *Away*. The analysis comparing the different Outcome groups revealed that the HR-ASD group exhibited less *smiles* compared to the HR-TD group. This might be because the contrast between the stimuli *Towards* and *Away* appeared unclear and more ambiguous than the other smiling and talking faces (stimuli *Face* and *Invariant*) and could have led to a reduced overall hedonic response for the HR-ASD group. We should note, however, that the HR-ASD smiles less in response to the more impoverished social response, in Condition 2, than the other groups, but more in response to being greeted by Hello, in Condition 1. Future studies will have to determine whether this reflects the higher familiarity with the later stimulus.

When the social stimulus was less predictable, in the third condition, contrary to all the other groups who showed significantly more *initial looks* and *longer looking* to the stimulus *Variable*, the HR-ASD group did not show a preference for either stimulus. We had initially predicted that HR-ASD would possibly prefer the *Invariant* stimulus. A preference for variable but relatively simple events in typical development is supported by previous studies showing that typically developing infants preferably orient towards neither too predictable or unpredictable, and neither too simple or too complex stimuli (Kidd et al., 2012). This suggests that there is an optimal level of prediction error that the learning brain most benefits from (Gottlieb et al., 2013). We offer partial support for the idea that there may be a shift in this optimal level, in ASD since this group did not show a preference for the *Invariant* stimulus. This may have resulted from limitations in assessing the predictability of stimuli, rather than in evaluating them. Condition 3 had a higher number of trials specifically because we wanted to give children sufficient evidence for the differences between the two stimuli – this may not have been sufficient for the HR-ASD group. However, we note that there was no effect of Trials, nor an interaction between Outcome and Trials, suggesting no change in preference through the experiment, in either group. Alternatively, rather than its unpredictability, the contents of interaction in the *Invariant* stimulus may have been unsatisfactory for the HR-ASD group. This condition alternated addressing the child with ‘Hello’, or ‘Good job’ or simply smiling towards her. Although all these stimuli were emotionally positive, it is possible that HR-ASD are mostly drawn to more prototypical ‘Hello’ stimulus (the one that elicits most smiles from all groups, as discussed above). A better controlled study would have compared the current *Variable* condition with 3 *Invariant* conditions, each depicting one of the stimulus types used in the *Variable* condition. Follow-up studies will have to address these confounding factors in search for more solid evidence for social interaction difficulties in ASD stemming from a preference for more predictable interaction.

Our evidence for typical social orienting and emotional engagement with social stimuli is in line with other studies on younger populations at risk (e.g. Elsabbagh et al., 2013). Some studies have suggested that orienting mechanisms may initially be typical, but that engagement with social cues gradually declines over the first two years of life, so that by toddlerhood, children with ASD look less to faces and eyes than control participants (Jones and Klin, 2013, Chawarska et al., 2010). The discrepancy with our study may result from our stimuli being particularly suited to encourage engagement, by presenting profile views of faces and only establishing eye contact when and if the child decided to do so. This contrast is relevant for the design of live or computerized interventions; some interventions or training programmes already build in the idea that interaction should be driven by the child with autism, rather than a parent or a teacher (Green et al., 2015, Bernardini et al., 2014). However, in contrast to typical orienting mechanisms, other work points to atypical processing of social information in the early development of infants that later receive a diagnosis of ASD (e.g. Elsabbagh et al., 2013, Jones et al., 2016). More studies of early development should take on the challenge of figuring out what about social information is difficult to process, whether it is its multimodal nature (e.g. Shic et al., 2014) or, as our findings suggest, the fact that it is driven by less transparent rules, which, unless understood, can look erratic and unpredictable.

## 5 Conclusion

In the current study, toddlers with ASD made initial saccade, reoriented their gaze, and smiled more towards a person addressing them than towards a toy. A typical reward-seeking behaviour and hedonic response towards social stimuli seem to be present in toddlers with ASD which goes against theories suggesting impaired social orienting or impaired motivation to engage with social stimuli. However, when presented with variable and invariant social stimuli, low-risk control and high-risk toddlers, who do not have ASD, selectively oriented their attention towards the variable interaction, while toddlers with ASD showed no preference for either stimulus. This lesser drive towards variable stimulation may reflect either a shift in the bias towards more predictable information or general difficulty with processing event statistics. To our knowledge, this is the first study assessing simultaneously the effect of the rewarding and variable nature of social stimuli in toddlers with and without ASD. This was made possible by using gaze-contingent eye-tracking, which allows the interaction to be controlled by the participants. This method will allow the finer manipulation of simulated social interaction, to further probe the condition under which impaired social interaction emerges in ASD.

## Conflict of Interest

None.

## Appendix A

The BASIS team in alphabetical order: Baron-Cohen, S.^3^, Bedford, R.^4^, Bolton, P.^4^, Cheung, H. M. C. ^1^, Davies, K. ^1^, Elsabbagh, M.^1^, Fernandes, J.^1^, Gammer, I.^4^, Guiraud, J.^1^, Liew, M.^1^, Maris, H.^1^, O’Hara, L.^1^, Pasco, G.^2^, Pickles, A.^4^, Ribeiro, H.^1^, Salomone, E.^2^, Tucker, L.^1^, Yemane, F.^1^.

^3^Autism Research Centre, University of Cambridge, UK.

^4^ NIHR Biomedical Research Centre, Kings College London, UK.
