# Supplementary material for: Simulating interaction: Using gaze-contingent eye-tracking to measure the reward value of social signals in toddlers with and without autism
Source: Dev Cogn Neurosci. 2017 Aug 12;29:21–9. doi: 10.1016/j.dcn.2017.08.004 (PMC6987892; doi:10.1016/j.dcn.2017.08.004)
Supplement: Supplementary file 1 [file mmc1.docx]

Supplementary online material (SOM).

1. Clinical measures and participant characteristics.
2. Analyses of second looks.
3. Effect of block and side bias.
4. Characteristics of the participants who contributed data to the analyses of smiles.
5. Analyses of the proportions of Initial looks and Smiles after including the factors Recruitment for intervention and Treatment.
6. Associations between task measures and clinical measures.
7. Clinical measures and participant characteristics.

Several behavioural assessments of social communicative and cognitive development were administered to the participants during the 27 and 36-month visits.

The *ASD Diagnostic Observation Schedule-Second Edition* (*ADOS-2*; [1]), a standardised observational assessment, was used to assess current symptoms of ASD (102 children were administered Module 2 and 14 participants Module 1, the ADOS was not completed by 2 LR participants during the 36-month visit). Calibrated Severity Scores for Social Affect, and Restricted and Repetitive Behaviours (RRB) were computed [2] to provide standardised ASD severity measures that account for differences in module administered, age and verbal ability.

The *Autism Diagnostic Interview-Revised* (*ADI-R*; [3], a structured parent interview, was completed by the parents of all the participants. Standard algorithm scores were computed for Reciprocal Social Interaction (Social), Communication, and Restricted, Repetitive and Stereotyped Behaviours and Interests (RRB). These assessments were conducted, without blindness to the risk-group status, by or under the close supervision of clinical researchers (i.e., psychologists, speech therapists, TC, GP). The *Social Communication Questionnaire* (SCQ; [4]) was used as additional parent-report measures of ASD symptoms.

The early learning composite score of the *Mullen Scales of Early Learning* (MSEL; [5]) was used to obtain a standardised measure of developmental abilities at 27 and 36 months of age.

Experienced clinical researchers (TC, GP) reviewed information on ASD symptomatology (ADOS-2, ADI-R, SCQ), adaptive functioning (*Vineland Adaptive Behavior Scale-II*, [6]), and development (MSEL) for each LR and HR child to ascertain ASD diagnostic outcome according to the DSM-5 [7]. None of the 26 LR children (14 boys, 12 girls) met the DSM-5 criteria for ASD and none had a community clinical ASD diagnosis. Of the 92 HR participants included in this paper, 14 participants (13 boys, 1 girl) met the criteria for a diagnosis of ASD (hereafter, HR-ASD). The remaining 78 HR participants did not meet the ASD criteria and were included in a second HR subgroup, (hereafter, HR-no ASD). This last group comprised 53 HR participants (24 boys, 29 girls) who were typically-developing and 25 HR participants (15 boys, 10 girls) who were not considered typically-developing, due to either a) scoring above the ADI-R cut-off for ASD [8] and/or scoring above the ADOS-2 cut-off for ASD (n =12), or b) scoring less than 1.5 SD below the population mean on the Mullen Early Learning Composite (< 77.5) or on the Mullen Expressive Language or Receptive Language subscales (< 35) (n =8), or meeting both of points a and b mentioned above (n =5).

1. Analyses of second looks.

To evaluate whether the participants reoriented their gaze towards the opposite stimulus after having previously triggered the activation of the first one with their initial look, second looks were entered in separate GEE analyses for each of the three conditions (Figure S1). Trials and Block were entered as within-subject factors and Outcome and Condition as a between-subject factors in the model. The Origin of the shift (the type of stimulus to which the participants first looked towards) was also included as a within-subject factor in the model. The analysis revealed a main effect of Condition (Waldχ2(2) = 6.81, p = .033, although follow up contrasts revealed no significant differences between the proportion of second looks between each condition). There was also a significant effect of Origin of shift (Waldχ2(1) = 72.92, p < .001) driven by a higher proportion of second looks from the non-prosocial stimuli (Toy, Away and Invariant) towards the prosocial stimuli (Face, Towards and Variable) but no significant effect of Outcome was found (Waldχ2(2) = 1.30, p = .522). The analysis also revealed two significant 2-way interactions between Condition and Outcome (Waldχ2(4) = 25.86, p < .001) and between Condition and Origin of shift (Waldχ2(2) = 148.87, p = < .001) but no other significant interactions were found (all p > .292). We followed-up on the significant Outcome * Condition interaction with three separate GEEs, one for each condition while also exploring interactions between the factors Outcome and Origin of shift in each condition as this was our main interest in this analysis. Condition 1: Face vs. Toy. There was a significant main effect of Origin of shift (the participants shifted more towards the *Face* when they first gazed at the *Toy* as opposed to the reverse shift, (Waldχ^2^(1) = 138.24, p < .001) and a significant main effect of Outcome (Waldχ^2^(2) = 10.95, p=.004) but follow up contrasts yielded no significant differences between the Outcome groups after Bonferroni corrections. There was no significant 2-way interaction between Outcome * Origin of shift (Waldχ^2^(2) = .23, p = .894). Additionally, the analyses revealed a main effect of Trials (a decrease in *second looks* over trials, Waldχ^2^(1) = 13.88, p < .001) and Block (an increase in *second looks* over block, Waldχ^2^(1) = 51.89, p < .001). *Condition 2: Towards vs. Away*. The analyses revealed no significant effect of Origin of shift (Waldχ^2^(1) = 2.61, p = .106), no significant effect of Outcome (Waldχ^2^(2) = 5.23, p = .073) and no significant 2-way interaction Outcome * Origin of shift (Waldχ^2^(2) = 1.47, p = .480). There was also no effect of Trials (Waldχ^2^(1) = .52, p=.470) nor Block (Waldχ^2^(1) = .20, p=.655). *Condition 3: Variable vs. Invariant*. There was no effect of Origin of shift (Waldχ^2^(1) = 1.09, p=.297), no main effect of Outcome (Waldχ^2^(2) = 4.28, p=.118) and no significant 2-way interactions Outcome * Origin of shift (Waldχ^2^(2) = .01, p=.997). There was a significant effect of Trials (a decrease in *second looks* over trials, Waldχ^2^(1) = 4.38, p=.036) and an effect of Block (a decrease in *second looks* over block, Waldχ^2^(1) = 4.77, p=.029).

Figure S1. Proportion of *second looks* (marginal means from the GEE analysis) towards the second stimulus after having previously looked at the first one, condition 1 (left panel), condition 2 (central panel) condition 3 (right panel). The proportion of *second looks* is plotted for each origin of shift, each condition and each group Outcome. Error-bars: +/- 1 standard error.

1. Effect of block and side bias.

A significant main effect of Block was thus revealed by the GEE analyses of *Initial looks* for each of the three conditions. More specifically, from the first to the second block, the analyses revealed a **decrease** in *Initial looks* towards the stimulus *Face*, an **increase** in *Initial looks* towards the stimulus Towards and a **decrease** in *Initial looks* towards the stimulus *Variable*. The location of the stimulus in each condition may explain this Block effect. In condition 1 and 3, the stimuli *Face* and *Variable* were displayed on the left side of the screen in the first block and on the right side in the second block. In condition 2, the stimulus *Towards* was displayed on the right side of the screen in the first block and on the left side in the second block. Therefore, the participants seem to show more *Initial looks* towards the stimuli displayed on the left side of the screen in the three conditions. This leftward attentional bias in selective orienting has already been described in the past [9] and several studies have investigated its possible cerebral origins [10,11]. Interestingly, exhibiting a left side bias is usually encountered in typical individuals and it has recently been suggested that a lack of left visual field bias might reveal actual deficits of attention, especially in face processing. This lack of leftward bias has been shown in adults with dyslexia [12] but also and importantly in regards to this current study, during facial processing in 6 and 11 month old infants with a high risk of autism [13], 2-5 year old children with autism [14] as well as adults with autism [15]. Indeed, in the current study, as already mentioned in the main text, two interactions between Outcome and Block were found. In the condition 1 (Waldχ^2^(2) = 6.05, p = .048), the interaction was driven by more *initial looks* toward the *Face* in Block 1 than in Block 2 for the LR (p = .001) and HR-no ASD (p < .001) groups but not for the HR-ASD group (p = 1.000). In condition 3 (Waldχ^2^(2) = 8.12, p = .017) the interaction was driven by a decrease in *initial looks* to the Variable stimulus from Block 1 to Block 2 for the HR-no ASD group only (p < .001). As we can see in Figure S2, the HR-ASD group showed a lesser change in orienting between block 1 and 2, across all conditions. Interestingly, the weaker Block effect in both Condition 1 and 3, for the HR-ASD group, suggests that the side bias is not necessarily driven by faces per se, but that the nature of the communicative content of the stimulation matters as well.

Figure S2. Proportion of *initial looks* (marginal means from the GEE analysis) towards the stimulus *Face* from the *Condition 1* (left panel), stimulus *Towards* from the *Condition 2* (central panel) and stimulus *Variable* from the *Condition 3* (right panel). The proportion of *initial looks* is plotted for both blocks and for all the groups separately. Error-bars: +/- 1 standard error.

1. Characteristics of the participants who contributed data to the analyses of smiles.

Table S2. Participant characteristics.

Abbreviations: ELC, Early Learning Composite; ADOS, Autism Diagnostic Observation Schedule; RRB, Repetitive and Restricted Behaviours; ADI-R, Autism Diagnostic Interview-Revised.

Significance of pairwise comparisons: ^a^ between the HR-ASD and LR groups, ^b^ between the HR-ASD and HR-no ASD groups and ^c^ between the HR-no ASD and LR groups.

1. Analyses of the proportions of Initial looks and Smiles while including the factors Recruitment and Treatment.

For all the analyses reported in the main manuscript, we began by including two binary terms Treatment (Treated vs Non-Treated) and Recruitment (Recruited for intervention vs Not recruited for intervention) as predictors. As we were not interested in investigating the effects of the intervention and recruitment, we only carried out this analysis to examine whether the inclusion of these factors would alter the significance of the results presented in the main manuscript.

*Analysis of Initial looks*. The *initial looks* were entered in a GEE analysis. The stimuli *Face* (condition 1), *Towards* (condition 2) and *Variable* (condition 3) were coded as 1 while the other corresponding choices were coded as 0. Condition, Trials, Block were entered as within-subject factors and Outcome (LR, HR-no ASD, HR-ASD), Treatment (Treated, Non-treated) and Recruitment (Recruited, Non-recruited) as between-subject factors in the model. The interaction between Outcome and Block became significant (Waldχ^2^(2) = 8.05, p = .018) as well as the 3-way interaction between Outcome, Condition and Block (Waldχ^2^(4) = 11.01, p = .026). However, follow up contrasts revealed no significant interaction between Outcome and Block in any of the different conditions (all p > .056). The analyses did not change the significance level of any of the other main effects and interactions of interest reported in the main manuscript.

*Analysis of Looking time*. A mixed ANOVA examining the proportion of looking time with the Outcome, Treatment and Recruitment as between-subject factors and the factor Condition (*Face* vs. *Toy*, *Towards* vs. *Away*, *Variable* vs. *Invariant*) as within-subject factor was conducted. The analyses did not change the significance level of any of the effects reported in the main manuscript.

*Analysis of Smiles.* Positive facial expressions categorised as *smiles* were entered in a GEE analysis. Condition, Trials and Block were entered as within-subject factors and Outcome, Treatment and Recruitment as between-subject factors in the model. The Type of stimulus that was triggered on each trial (*Face*, *Towards* and *Variable* vs. *Toy, Away* or *Invariant*) was also included as a within-subject factor in the model. The analyses did not change the significance level of any of the effects and interactions reported in the main manuscript.

1. Associations between task measures and clinical measures.

In order to investigate whether the *initial looks, looking time* and *smiles* were related to developmental functioning and/or autistic traits, correlational analyses of the proportions of *initial looks, looking time* and *smiles* with the early learning composite scores of the Mullen Scales as well as the sub-scores of the ADOS *(*ADOS-social affect and ADOS-repetitive and restricted behaviours (RRB) calibrated severity scores) and the ADI algorithm scores at the 27-month and 36-month visits were computed for each of the conditions. No significant correlations were found after Bonferroni corrections.

References.

[1] Lord C, Rutter M, DiLavore P, Risi S, Gotham K, Bishop S. Autism Diagnostic Observation Schedule--2nd edition (ADOS-2), 2012.

[2] Gotham K, Pickles A, Lord C. Standardizing ADOS Scores for a Measure of Severity in Autism Spectrum Disorders. J Autism Dev Disord 2009;39:693–705. doi:10.1007/s10803-008-0674-3.

[3] Le Couteur A, Lord C, Rutter M. The autism diagnostic interview-revised (ADI-R), 2003.

[4] Rutter Bailey, A., & Lord, C. M. Social Communication Questionnaire. Western Psychological Services; 2003.

[5] Mullen EM. Mullen scales of early learning. Circle Pin. MN: American Guidance Service, Inc.: Circle Pines, MN: American Guidance Service, Inc.; 1995.

[6] Sparrow SS, Cicchetti D V, Balla DA. Vineland adaptive behavior scales, Second edition (Vineland II). Circle Pines, MN: American Guidance Service, Inc.; 2005. doi:10.1002/9780470373699.

[7] American Psychiatric Association. Diagnostic and Statistical Manual of Mental Disorders (DSM-5®). 2013. doi:10.1176/appi.books.9780890425596.744053.

[8] Risi S, Lord C, Gotham K, Corsello C, Chrysler C, Szatmari P, et al. Combining information from multiple sources in the diagnosis of autism spectrum disorders. J Am Acad Child Adolesc Psychiatry 2006;45:1094–103. doi:10.1097/01.chi.0000227880.42780.0e.

[9] Kinsbourne M. The cerebral basis of lateral asymmetries in attention. Acta Psychol (Amst) 1970;33:193–201. doi:10.1016/0001-6918(70)90132-0.

[10] Thomas NA, Castine BR, Loetscher T, Nicholls MER. Upper visual field distractors preferentially bias attention to the left. Cortex 2014;64:179–93. doi:10.1016/j.cortex.2014.10.018.

[11] Okon-Singer H, Podlipsky I, Siman-Tov T, Ben-Simon E, Zhdanov A, Neufeld MY, et al. Spatio-temporal indications of sub-cortical involvement in leftward bias of spatial attention. Neuroimage 2011;54:3010–20. doi:10.1016/j.neuroimage.2010.10.078.

[12] Hari R, Renvall H, Tanskanen T. Left minineglect in dyslexic adults. Brain 2001;124:1373–80.

[13] Dundas E, Gastgeb H, Strauss MS. Left Visual Field Biases when Infants Process Faces: A Comparison of Infants at High- and Low-Risk for Autism Spectrum Disorder. J Autism Dev Disord 2012;42:2659–68. doi:10.1007/s10803-012-1523-y.

[14] Guillon Q, Hadjikhani N, Baduel S, Kruck J, Arnaud M, Rogé B. Both dog and human faces are explored abnormally by young children with autism spectrum disorders. Neuroreport 2014;25:1237–41. doi:10.1097/WNR.0000000000000257.

[15] Dundas EM, Best CA, Minshew NJ, Strauss MS. A Lack of Left Visual Field Bias When Individuals with Autism Process Faces. J Autism Dev Disord 2012;42:1104–11. doi:10.1007/s10803-011-1354-2.
